# DEGRO guideline for personalized radiotherapy of brain metastases and leptomeningeal carcinomatosis in patients with breast cancer

**DOI:** 10.1007/s00066-024-02202-0

**Published:** 2024-03-15

**Authors:** Kai J. Borm, Sophie T. Behzadi, Juliane Hörner-Rieber, David Krug, Rene Baumann, Stefanie Corradini, Marciana Nona Duma, Jürgen Dunst, Gerd Fastner, Petra Feyer, Rainer Fietkau, Wulf Haase, Wolfgang Harms, Thomas Hehr, Christiane Matuschek, Marc D. Piroth, Leonard Christopher Schmeel, Rainer Souchon, Vratislav Strnad, Wilfried Budach, Stephanie E. Combs, Breast Cancer Expert Panel of the German Society of Radiation Oncology DEGRO

**Affiliations:** 1 TUM School of Medicine, Department of Radiation Oncology, Technical University of Munich, Ismaninger Straße 22, 81675 Munich, Germany; 2 Department of Radiation Oncology, Heidelberg University Hospital, Heidelberg, Germany; 3 Department of Radiation Oncology, University Hospital Schleswig-Holstein, Kiel, Germany; 4 Department of Radiation Oncology, St. Marien-Krankenhaus, Siegen, Germany; 5 Department of Radiation Oncology, University Hospital, LMU Munich, Munich, Germany; 6 Department of Radiation Oncology, Helios Clinics of Schwerin—University Campus of MSH Medical School Hamburg, Schwerin, Germany; 7 Department for Human Medicine, MSH Medical School Hamburg, Hamburg, Germany; 8 Department of Radiotherapy and Radio-Oncology, University Hospital Salzburg, Landeskrankenhaus, Paracelsus Medical University, Salzburg, Austria; 9 Formerly Department of Radiation Oncology, Vivantes Hospital Neukölln, Berlin, Germany; 10 Department of Radiation Oncology, University Hospital Erlangen, Erlangen, Germany; 11 Formerly Department of Radiation Oncology, St.-Vincentius-Hospital Karlsruhe, Karlsruhe, Germany; 12 Department of Radiation Oncology, St. Claraspital, Basel, Switzerland; 13 Department of Radiation Oncology, Marienhospital Stuttgart, Stuttgart, Germany; 14 Department of Radiation Oncology, University Hospital Düsseldorf, Düsseldorf, Germany; 15 Department of Radiation Oncology, HELIOS University Hospital Wuppertal, Witten/Herdecke University, Wuppertal, Germany; 16 Department of Radiation Oncology, University Hospital Bonn, Bonn, Germany; 17 Formerly Department of Radiation Oncology, University Hospital Tübingen, Tübingen, Germany; 18 Partner Site Munich, Deutsches Konsortium für Translationale Krebsforschung (DKTK), Munich, Germany; 19 Department of Radiation Medicine (IRM), Helmholtz Zentrum München (HMGU), Neuherberg, Germany

**Keywords:** Molecular profile, Radiosurgery, Whole-brain radiotherapy, Craniospinal irradiation, Neurocognitive side effects

## Abstract

**Purpose:**

The aim of this review was to evaluate the existing evidence for radiotherapy for brain metastases in breast cancer patients and provide recommendations for the use of radiotherapy for brain metastases and leptomeningeal carcinomatosis.

**Materials and methods:**

For the current review, a PubMed search was conducted including articles from 01/1985 to 05/2023. The search was performed using the following terms: (brain metastases OR leptomeningeal carcinomatosis) AND (breast cancer OR breast) AND (radiotherapy OR ablative radiotherapy OR radiosurgery OR stereotactic OR radiation).

**Conclusion and recommendations:**

Despite the fact that the biological subtype of breast cancer influences both the occurrence and relapse patterns of breast cancer brain metastases (BCBM), for most scenarios, no specific recommendations regarding radiotherapy can be made based on the existing evidence. For a limited number of BCBM (1–4), stereotactic radiosurgery (SRS) or fractionated stereotactic radiotherapy (SRT) is generally recommended irrespective of molecular subtype and concurrent/planned systemic therapy. In patients with 5–10 oligo-brain metastases, these techniques can also be conditionally recommended. For multiple, especially symptomatic BCBM, whole-brain radiotherapy (WBRT), if possible with hippocampal sparing, is recommended. In cases of multiple asymptomatic BCBM (≥ 5), if SRS/SRT is not feasible or in disseminated brain metastases (> 10), postponing WBRT with early reassessment and reevaluation of local treatment options (8–12 weeks) may be discussed if a HER2/Neu-targeting systemic therapy with significant response rates in the central nervous system (CNS) is being used. In symptomatic leptomeningeal carcinomatosis, local radiotherapy (WBRT or local spinal irradiation) should be performed in addition to systemic therapy. In patients with disseminated leptomeningeal carcinomatosis in good clinical condition and with only limited or stable extra-CNS disease, craniospinal irradiation (CSI) may be considered. Data regarding the toxicity of combining systemic therapies with cranial and spinal radiotherapy are sparse. Therefore, no clear recommendations can be given, and each case should be discussed individually in an interdisciplinary setting.

## Introduction

Breast cancer is the leading cause of cancer-related death in women worldwide [1]. However, due to advances in medical treatment, mortality rates have been declining in western countries in the last few decades [1].

Despite good extracranial disease control, physicians are increasingly faced with long-term survivors developing brain metastases or leptomeningeal spread [2]. Patients with breast cancer brain metastases (BCBM) require special attention, as adequate treatment can lead to improved prognosis and long-term survival [3, 4].

The reported incidence of brain metastases in breast cancer patients ranges from 5.1% in patients with newly diagnosed breast cancer to up to 33% in patients with metastatic breast cancer [5–7]. Asymptomatic breast cancer patients are not routinely screened for brain metastases with magnetic resonance imaging (MRI). Hence, BCBM are mostly diagnosed due to emerging neurologic symptoms and the actual incidence of BCBM is expected to be even higher [8]. The median overall survival after diagnosis of BCBM ranges from 3 to > 26 months [2]. However, prognosis of patients with BCBM depends on several factors such as biological subtype, age, general condition, number and size of brain metastases, and control of systemic disease [7, 9–11].

Traditionally, local therapies such as neurosurgery and radiotherapy were considered as the only treatment option for BCBM due to the limited effectiveness of systemic therapies due to the blood–brain barrier. However, newer systemic therapies such as HER2-targeted therapies have shown promising central nervous system response rates. Nonetheless, local control rates achieved with radiotherapy (with or without resection) remain unrevealed [12, 13].

The appropriate use of radiation techniques such as stereotactic radiosurgery (SRS), fractionated stereotactic radiotherapy (SRT), whole-brain radiotherapy (WBRT), or craniospinal irradiation (CSI) is essential for the oncological treatment of patients and preservation of quality of life. However, with the increasing central nervous system (CNS) response rates of especially HER2-targeted therapies, there is a need to reassess the current literature to make evidence-based recommendations.

## Methods

For the current review, a PubMed search was conducted including articles from 01/1985 to 05/2023. The search was performed using the following terms: (brain metastases OR leptomeningeal carcinomatosis) AND (breast cancer OR breast) AND (radiotherapy OR ablative radiotherapy OR radiosurgery OR stereotactic OR radiation). We report results of reviews, randomized trials, and high-quality retrospective studies.

## Results

### Prognosis of BCBM in dependence of biological subtype

The incidence of BCBM in HER2+ and triple-negative breast cancers (TNBC) is twice as high compared to hormone receptor (HR)-positive HER2− tumors [7]. Furthermore, patients with HR+ breast cancer have a significantly longer median interval until the development of brain metastases [14]. While BCBM in TNBC occur often in case of systemic/extracranial disease progression, in HER2+ tumors, BMBC develop more frequently without evidence of extracranial disease progression. There are several prognostic scores for breast cancer patients with brain metastases. In 2012, Sperduto and Kased et al. [15] published a summary report on the graded prognostic assessment (GPA) for patients with brain metastases, which was updated in 2020. The prognostic factors in breast GPA are tumor subtype, general condition (Karnofsky performance score), age, presence of extracranial disease, and number (1 vs. > 1) of brain metastases (Sperduto, Mesko et al. 2020). Luminal A/B and HER2+ tumors are associated with a significantly better outcome compared to TNBC, and patients with a single brain metastasis had superior survival compared to patients with multiple BCBM [16]. However, there was no further prognostic stratification according to the number of brain metastases for patients with more than one BCBM.

HR+/HER2− tumors can be associated with a better intracranial control rate after brain-directed radiotherapy (WBRT, SRS, or SRT) in comparison to HER2+ and TNBC brain metastases [14]. While HER2+ brain metastases are associated with a lower local control, TNBC are at higher risk of developing distant recurrences in the previously untreated brain [17]. Nevertheless, local treatment including surgery and SRS significantly improves local control rates in both HER2+ and triple-negative BCBM [18].

The microenvironment in the CNS differs from the breast and other extracranial sites [19]. Hence, breast cancer tumor cells that have the ability to cross the brain–blood barrier and proliferate effectively in the CNS need specific characteristics, which are not yet fully understood. Interestingly, in earlier studies, a discordance between primary tumor and BCBM receptors of > 40% was reported (Sperduto, Mesko et al. 2020, [9]). This further impedes treatment decisions based on molecular subtype but also emphasizes the value of local treatment as the biological subtype of BCBM often remains unknown and systemic therapies may not be adapted to the biological subtype of brain metastases (Table 1).

**Table 1:** Impact of biological subtype on incidence and prognosis in BCBM

Study	Design	Aim	Study collective	Results
*Incidence*				
Kuksis et al. 2021 [7]	Systematic Review	Incidence of BCBM among metastatic BC stratified by molecular subtype	Number of BC Patients: HR+/HER2−: 14,656 HER2+: 5971 TNBC: 4102	Cumulative incidence of BMBC: HR+/HER2−: 15% HER2+: 31% TNBC: 32%
*Prognosis*				
Sperduto et al. 2020 [2]	Multicenter retrospective analysis	Breast prognostic index in survival in breast cancer brain metastases	Patients with BCBM: Luminal A: 772 Luminal B: 527 (REF) TNBC: 595 HER2+: 421	Worse survival associated with luminal A (HR 2.0) and TNBC (HR 2.8)
Kim et al. 2020 [20]	Multicenter retrospective analysis	Prognostic factors regarding overall survival in BCBM	730 patients with BCBM	Median FU: 11.9 months HR- and HER2- were associated with inferior OS in multivariate analyses
Kim et al. 2021 [14]	Multicenter retrospective analysis	Impact of molecular subtype on distant intracranial control after WBRT, SRS, or SRT for BCBM	Patients with BCBM HR+/HER2−: 136 HER2+: 253 TNBC: 149	1‑year intracranial distant control: HR+/HER2−: 48.1% HER2+: 56.0% TNBC: 30.4% Anti-HER2 therapy and initial use of WBRT significantly lowered rate of new BCBM
Cagney et al. 2019 [17]	Monocentric retrospective analysis	Impact of molecular subtype on intracranial control after WBRT, SRS or SRT	Patients with BCBM: HR+/HER2−: 116 HER2+: 164 TNBC: 69	2‑year intracranial local control: HR+/HER2−: 82.2% HER2+: 79.1% TNBC: 50.8% Intracranial distant control: Compared to HR+/HER2−: HER2+: HR 1.01 (*p* = 0.97) TNBC: HR: 3.2 (*p* < 0.001)
Chong et al. 2015 [18]	Multicenter retrospective analysis	Impact of molecular subtype on treatment outcome (surgery/SRS + WBRT vs. WBRT alone)	Patients with BCBM HR+/HER2−: 29 HER2+: 47 TNBC: 40	Median FU: 50.9 months HR+/HER2− had the best, TNBC the worst OS and local control rates. Local treatment (SRS/Surgery) in addition to WBRT improved intracranial control rates in HER2+ and TNBC subtype

### Radiotherapy in BCBM

The randomized studies found in literature on radiotherapy for brain metastases mostly included patients with various tumor entities and were not specifically focused on breast cancer. Even though breast cancer is the second most common primary tumor type in patients with brain metastases [21] and a significant proportion of patients included in the randomized studies had breast cancer (Tables 2 and 3), specific conclusions for the subgroup of breast cancer patients or even for individual molecular subtypes are difficult to draw. Therefore, the following statements and recommendations are based on the general literature for radiotherapy of brain metastases.

**Table 2:** Selected studies investigating the role of SRS, WBRT, and surgery in limited BCBM

Study	Design	Study collective/proportion of BCBM	Results
*SRS/SRT (+/− WBRT) for intact BM*			
Redmond et al. 2021 [22] (HyTEC)	TCP models based on pooled dosimetric and clinical data from 56 studies	Total number of BM 13,929 BCBM among the 4 most frequent entities	SRS for BM ≤ 20 mm with 18 and 24 Gy corresponded to > 85% and 95% 1‑year LC rates, respectively. SRT (3–5 fx) in the range of 27 to 35 Gy, 80% 1‑year LC was achieved for tumors of 21 to 40 mm in diameter
Andrews et al. 2004 [23] (RTOG 9508)	Randomized multicenter trial Arms: 1) WBRT vs. 2) WBRT + SRS	333 patients with 1–3 BM 34 patients (10.2%) with BCBM	Survival benefit in 1 BM (median survival 6.5 vs. 4.9 months) for the SRS + WBRT arm
Kocher et al. 2011 [24] (EORTC 22952-26001)	Randomized phase III multicenter trial Arms: SRS or resection + 1) WBRT vs. 2) Obs	359 patients with 1–3 BM 42 patients (12%) with BCBM	No difference in median OS (10.9 vs. 10.7 months) WBRT improved both 2‑year intracranial local control (69% vs. 81%) and 2‑year intracranial distant control (52% vs. 67%)
Churilla et al. 2019 [13]	Unplanned exploratory analysis of EORTC 22952–26001: comparison of intracranial local control of SRS vs. resection	268 patients with 1–2 BM 28 patients (10.4%) with BCBM	Intracranial local control after 1 year: 82% resection vs. 86.8% SRS. HR 1.15 SRS associated with improved early local control compared with surgery. → No differences between SRS and surgery as primary treatment
Brown et al. 2016 [25]	Randomized multicenter phase III trial Arms: SRS +1) WBRT vs. 2) obs	213 patients with 1–3 BM 18 patients (8.4%) with BCBM	Less cognitive deterioration and improved quality of life with SRS alone compared to SRS + WBRT 1‑year intracranial local control: 90.4% (SRS + WBRT) vs. 72.6% (SRS) (*p* < 0.05) Intracranial distant control: 92.5% (SRS + WBRT) vs. 70% (SRS) (*p* < 0.05) Median OS 10.4 (SRS) vs. 7.4 months (SRS + WBRT; HR 1.02; *p* = 0.92)
Aoyama 2006 [12]	Randomized controlled multicenter trial Arms: 1) WBRT + SRS vs. 2) SRS	132 patients with 1–4 BM, 9 patients (6.8%) with BCBM	No difference in median OS (7.5 vs. 8.0 months) Additional WBRT improved intracranial distant control after 1 year (recurrence rate 46.8% for SRS + WBRT vs. for 76.4% SRS)
Wijetunga 2020 [26]	Randomized controlled trial. Arms: 1) SRS + WBRT vs. 2) SRS	58 patients with 1–3 BM, 8 patients (13.8%) with BCBM	Intracranial distant control at 1 year 73% (SRS + WBRT) vs. 27% (SRS) Mean probability of cognitive dysfunction at 4 months in 52% (SRS + WBRT) vs. 24% (SRS)
*Postoperative SRS for resected BM*			
[27]	Single center trial Postoperative SRS	79 patients with 1–4 BM	Median PFS 10.0 months, median OS 14.3 months. 1‑year local intracranial control: 89.8%
Kayama 2018 [28]	Noninferiority randomized controlled phase III trial Arms: postoperative WBRT vs. salvage SRS to cavity	271 patients with 1–4 BM, 53 patients (19.5%) with BCBM	No difference in median OS (15.6 months). Improved intracranial PFS after WBRT (10.4 months) vs. salvage SRS (4.0 months) with increased toxicity (grade II–IV cognitive dysfunction in 16.4% vs. 7.7%)
Mahajan et al. 2017 [29]	Randomized controlled trial Arms: 1) Observation vs. 2) postoperative SRS	132 patients with 1–3 BM, 23 patients (17.4%) with BCBM	12-month local tumor control in BCBM 50% SRS with a HR of 0.5 of local control (*p* < 0.05)
Brown 2017 [30]	Randomized controlled phase III trial Arms: 1) postoperative WBRT vs. 2) postoperative SRS	194 patients with 1 resected BM; < 5.0 cm	Significantly worse local and distant brain control in SRS group compared to WBRT. No difference in OS
Eitz 2020 [31]	Retrospective multicenter cohort study of postoperative SRT	558 patients with resected BM. 101 patients with BCBM (17.6%)	Local control was 94% at 5 months, 84% at 1 year, 75% at 2 years, and 71% at 3 years (median was not reached)

**Table 3:** Selected studies investigating the role of SRS in multiple brain metastases

Study	Design	Study collective/proportion of BCBM	Results
*SRS in multiple BM*			
Chang et al. 2010 [32]	Single-center retrospective analysis	323 patients with BM: Group 1: 215 patients with 1–5 BM Group 2: 58 patients with 6–10 BM Group 3: 17 patients with 11–15 BM Group 4: 33 patients with > 15 BM	No significant differences in intracranial local control rates, however, group 4 showed the worst intracranial distant control No significant differences in median OS: Group 1: 10 months Group 2: 10 months Group 3: 13 moths Group 4: 8 months
Yamamoto 2014 [33] (JLGK0901)	Multicenter prospective observational study	1194 patients with 1-10 BM, 455 patients with 1 BM, 531 patients with 2-4 BM, 208 patients with 5-10 BM. 123 patients (10%) with BCBM	No significant difference in OS for patients with 5–10 BM compared to patients with 2–4 BM Median OS: 1 BM: 13.9 months 2–4 BM: 10.8 months 5–10 BM: 10.8 months No significant differences in local recurrence during follow-up
Wilson et al. 2020 [34]	Single-center retrospective analysis	91 patients with 1–21 BCBM receiving SRS	Median OS 15.7 months Subgroups: ER+/HER2− 13.8 months ER+/HER2+ 21.4 months ER−/HER2+ 20.4 months TNBC 8.5 months Metastatic volumes > 10 cm^3^ were associated with significantly worse OS
*SRS in comparison to WBRT for multiple BM*			
El Shafie 2020 [35]	Retrospective matched-pair analysis	128 patients with 3–16 BM, 64 patients treated with SRS, 64 matched patients treated with WBRT. 24 patients (18.8%) with BCBM	Prolonged median OS (15.7 months vs. 8.0 months) and 1‑year intracranial local control 91.7% after SRS. Prolonged median intracranial (local and distant) control after WBRT (8.6 months SRS vs. 22.4 months WBRT)
Hartgerink, Bruynzeel et al. 2021 [36]	Phase III randomized multicenter trial Arms: 1) WBRT vs 2) SRS/SRT	29 patients with 4–10 BM, 1 patient (WBRT, 3.4%) with BCBM	No statistically significant differences due to premature closure of trial. 1‑year OS: 31% (WBRT) vs. 57% (SRS). QoL decrease in WBRT worse compared to SRS
J. Li 2020 [37]	Phase III randomized trial Arms: SRS vs. WBRT	72 patients with 4–15 BM, 36 patients treated with SRS, 36 patients treated with WBRT	SRS with reduced risk of neurocognitive deterioration. Improved median OS after SRS (10.4 vs. 8.4 months). Preliminary analysis of intracranial local control at 4 months LC 100% (SRS) vs. 95.5% (WBRT)
Kim, Kim et al. 2023 [38]	Multicenter retrospective analysis	471 patients with 1–10 BCBM 1–4 BCBM: *n* = 337 5–10 BCBM: *n* = 134	79.9 % of patients with ≥5 BCBM were treated with WBRT and only 9.0 with SRS In patients with ≤10 BCBM the number of BCBM and WBRT did not affect OS.TNBC/extracranial disease decreased OS TNBC/extracranial disease decreased OS

### Limited brain metastases in breast cancer (≤ 4)

The definition of “limited brain metastases” differs among studies and recommendations (Table 2). Most studies focusing on local therapy in limited brain metastases included patients with up to four metastases.

In randomized trials, surgery followed by WBRT for single brain metastases resulted in an overall survival benefit with fewer local recurrences compared to WBRT alone [39–41]. Hence, for many years, surgery has been considered as the preferred treatment option for these patients [42]. However, a subgroup analysis of RTOG 9508 also demonstrated an improvement in overall survival for patients with 1–3 brain metastases and a high GPA when SRS was added to WBRT [23, 43]. Further studies that have applied more modern imaging and treatment techniques have demonstrated that SRS and surgery have similar outcomes [13, 24]. On the other hand, WBRT after surgery or SRS did not show any improvement in quality of life or overall survival [24, 44]. Therefore, SRS has increasingly gained importance.

SRS is less invasive than surgery and generally well tolerated [42, 45]. According to a tumor control probability (TCP) model based on pooled data of > 56 manuscripts, local 1‑year control rates of up to 95% are achievable with SRS only [22]. Meanwhile, based on a randomized phase III trial, the addition of WBRT to SRS does not improve overall survival for patients with 1–3 brain metastases despite a reduced intracranial recurrence rate [12, 25, 46]. Given the reduced neurocognitive function and quality of life and lack of an overall survival benefit, radiosurgery alone should be considered as standard of care for 1–4 brain metastases [24–26, 46].

In certain cases, surgery might be necessary for brain metastases, such as for immediate decompression in the presence of symptomatic brain metastases, or for large metastases that are not suitable for SRS/SRT. Surgery may also be recommended for histological verification, particularly after a long recurrence-free interval. After surgery, postoperative radiotherapy is generally indicated to improve local control rates. In a randomized phase III trial, postoperative SRS (or SRT) was shown to have the same overall survival but improved neurocognitive function compared with postoperative WBRT [28, 30]. Therefore, if postoperative radiation is planned, SRS or SRT should be favored whenever possible with respect to tumor volume.

In case of limited brain metastases, SRS/SRT can be generally be recommended regardless of molecular subtype and systemic therapy (see sections “Combination of radiotherapy with systemic therapy” and “Recommendations”). However, for SRS/SRT in TNBC, a higher intracranial distant recurrence rate should be taken into account, indicating the necessity of close follow-up.

### Local therapy of multiple (≥ 5) brain metastases

For patients with multiple (≥ 5) brain metastases, WBRT has been the standard of care over the past decades [47]. In historical trials, it demonstrated a reduction of neurological symptoms and complications as well as an improvement in PFS compared to best supportive care [48]. However, WBRT also carries the risk of significant additional neurocognitive impairment during the remaining lifetime [48]. Up to two thirds of patients undergoing WBRT suffer from neurocognitive deterioration 2–6 months after treatment [49]. Although associated with less neurocognitive impairment, the value of using SRS in patients with more than four metastases has been discussed controversially [34, 48]. Recently, however, studies have demonstrated equivalence in overall survival (OS) when comparing patients with 2–4 and multiple (5–10) metastases after SRS [50]. Li et al. compared SRS to WBRT in a randomized manner including 72 patients with 4–15 brain metastases [37]. Even though SRS was associated with significantly worse intracranial distant control, no difference in OS and inferior cognitive function after WBRT was observed. A Dutch randomized phase III trial comparing SRS to WBRT in 4–10 BM was closed early due to poor accrual [51]. Observational studies suggest that patients with 5–10 metastases have similar outcomes compared to patients with 2–4 metastases after SRS [32, 33]. The treatment-related toxicity was low, with neurocognitive function being similar between the groups when cumulative tumor volume was < 15 ml [52]. In a retrospective multicenter analysis of 471 patients with 1–10 BCBM, the number of BCBM and the use of WBRT had no impact on OS [38].

Thus, SRS can be recommended as an efficient and safe treatment option even for multiple BCBM. Even in the case of in-field recurrence after prior SRS, a second course of SRS is often feasible with acceptable toxicity, and can be considered as a salvage treatment option for selected patients with good performance status and a time interval of > 12 months to initial SRS [53, 54].

In selected cases of HER2+ disseminated brain metastases and/or if SRS/SRT is technically infeasible, WBRT can be deferred with early follow-up and reevaluation of local treatment (see sections “Combination of radiotherapy with systemic therapy” and “Recommendations”).

### Focal radiotherapy and craniospinal axis irradiation in leptomeningeal carcinomatosis

Leptomeningeal tumor involvement represents a very advanced tumor stage with numerous complications [55]. In recent years, the incidence of leptomeningeal carcinomatosis (LC) in breast cancer has increased sharply, mainly due to improved treatment and thus a prolonged survival of patients [56]. Due to the difficulty of diagnosis, it is likely that the reported incidence of approximately 5% in breast cancer patients is an underestimation [57]. Compared with parenchymal involvement of the central nervous system, patients with leptomeningeal carcinomatosis generally have a worse prognosis [58]. Median overall survival is 4–6 weeks when untreated and reportedly up to 30.3 weeks when treated. However, the treated and untreated patients are difficult to compare and selection bias may play an important role [56, 59, 60]. Diffuse leptomeningeal spread should be differentiated from nodular involvement, which may occur more frequently after resection of brain metastases treated with postoperative SRS/SRT compared to WBRT and is more common in patients with BCBM compared to other entities [61, 62]. It remains uncertain whether the two types of leptomeningeal carcinomatosis require distinct treatment approaches, and whether the recently proposed new approach of preoperative stereotactic radiosurgery/stereotactic radiation therapy can effectively prevent the occurrence of nodular leptomeningeal carcinomatosis after surgery. Several ongoing trials aim to address these questions [63].

The work-up and treatment options for leptomeningeal carcinomatosis are summarized in the EANO-ESMO guideline on leptomeningeal metastasis [64].

To date, despite the existence of guidelines, treatment concepts still vary widely between centers and physicians [65], as scientific evidence is sparse [56, 66]. Possible treatment options for leptomeningeal carcinomatosis include intrathecal chemotherapy, radiotherapy, systemic therapy, and best supportive care.

Intrathecal chemotherapy is a controversial therapeutic approach for leptomeningeal carcinomatosis [55, 67–69] and is associated with severe side effects. The evidence regarding oncologic benefit is weak and based only on retrospective observational studies from more than 20 years ago, and is considered controversial in more recent studies, particularly with regard to OS [56, 59, 66, 70]. In contrast, systemic therapy has been shown to improve overall survival from 2 to 6 months [66, 70]. Recent data from small phase I/II studies suggest that the use of intrathecal trastuzumab potentially improves the outcomes for HER2-positive breast cancer patients with leptomeningeal disease [71, 72].

Another therapeutic option is radiotherapy [55]), which can be applied as involved-field irradiation (WBRT vs. spinal IFRT) to improve neurological systems and prevent complication, or as craniospinal irradiation (CSI), which additionally aims to improve the oncologic outcome by eradicating tumor cells in the entire craniospinal axis [73]. For selected patients with isolated nodular lesions, SRS/SRT may represent an option [74].

In 2022, the results of a randomized phase II trial (*n* = 62 patients) were published evaluating the oncological benefit of CSI [75]. The study enrolled patients with leptomeningeal carcinomatosis from solid tumors. A total of 63 patients were enrolled, of whom 43% had breast cancer. They were randomized 2:1 to receive either proton CSI (pCSI) or photon involved-field radiotherapy (IFRT). The primary endpoint was central nervous system progression-free survival (CNS PFS), with secondary endpoints including overall survival and treatment-related adverse events (TAEs). The results showed significant benefits regarding CNS PFS and OS after pCSI compared to IFRT, with no increase in serious TAEs. The findings of this trial reveal the potential of CSI in patients with leptomeningeal metastasis. Nevertheless, it should be noted that proton therapy for CSI is very rarely available, and the more widely available option of photon therapy results in a higher dose exposure to the bone marrow and other organs at risk (Table 4).

**Table 4:** Selected studies investigating the role of radiotherapy or intrathecal therapy in patients with leptomeningeal carcinomatosis in breast cancer

Study	Design	Study collective/proportion of BCBM	Results
*RT in LC*			
Rudnicka 2007 [73]	Single-center retrospective study on efficacy of multimodal treatment with IT chemotherapy, systemic therapy, and radiotherapy (WBRT/CSI)	67 patients with LC in breast cancer	Clinical response in 76% Median OS 16 weeks. In multivariate analysis, survival benefit for systemic therapy and IT chemotherapy (not for WBRT)
Yang 2022 [75]	Single-center phase II randomized trial Arms: proton CSI (pCSI) vs. involved-field RT (IFRT)	63 patients with LC, 42 patients treated with pCSI, 21 patients treated with IFRT	Significant benefit in CNS PFS with pCSI (7.5 months vs. 2.3 months) and OS (9.9 months vs. 6.0 months) at planned interim analysis. No difference in grade III/IV toxicity

*WBRT* whole brain radiotherapy, *CSI* craniospinal irradiation, *IT* intrathecal, *LC* leptomeningeal carcinomatosis, *OS* overall survival, *pCSI* proton craniospinal irradiation, *IFRT* involved-field radiotherapy, *CNS PFS* central nervous system progression-free survival

### Irradiation techniques and follow-up

The optimal use of SRT, SRS, and WBRT is discussed in detail in the EANO-ESTRO and ASTRO guidelines as well as in the HyTEC recommendations for the treatment of brain metastases [22, 76, 77].

The question of the optimal fractionation regimen is dependent on the size and location of the metastases/resection cavity. For smaller lesions (≤ 20–25 mm), SRS is usually performed with a dose ranging between 18 and 24 Gy prescribed to the 60–80% isodose. In larger metastases (> 20–25 mm) or in critical locations, SRT is typically given in 3–7 fractions with a total dose of 27–35 Gy prescribed to the 60–80% isodose.

Depending on the irradiation technique and the location of the metastases, prescription and normalization can differ (e.g., Gamma Knife with prescription to lower isodoses).

The total dose of WBRT is typically 30 Gy given in 10 fractions (prescribed to the median dose). In frail patients, a dose regimen of 20 in 5 fractions can be used. The use of N‑methyl-D-aspartate (NMDA) receptor antagonists such as memantine in combination with hippocampal avoidance (HA) has been shown to improve cognitive function, particularly memory, in patients undergoing WBRT [78]. Several studies have demonstrated that HA during WBRT with or without memantine or donepezil (acetylcholinesterase selective inhibitor) can effectively reduce the risk of cognitive decline in patients without compromising tumor control [79–82]. It should be noted that in Germany, the use of donepezil and memantine is off label and patients need to be informed about potential side effects such as blurred vision, dizziness, and headache (Table 5).

**Table 5:** Selected studies investigating the role of HA-WBRT/toxicity reduction

Study	Design	Study collective/proportion of BCBM	Results
*Donepezil/memantine*			
Rapp, Case et al. 2015 [83]	Phase III randomized trial “Brain tumor survivors” after WBRT Arms: 1) donepezil, 2) placebo	66% with primary brain tumors 27% with brain metastases	Significant differences favoring donepezil for memory, motor speed, and dexterity
Brown et al. 2013 [84]	Multicenter randomized, double-blind controlled study Arms: 1) WBRT + memantine, 2) WBRT + Placebo	554 patients with BM, 75 patients (13.5%) with BCBM 256 patients received memantine, 252 patients received placebo	No significant differences regarding cognitive decline in patients treated with memantine at 24 weeks, but significant differences favoring memantine in MMSE (Mini Mental State Examination) at 24 weeks
*Hippocampal avoidance during WBRT*			
Gondi et al. 2014 [80] (RTOG 0933)	Multicenter single-arm phase II study on HA-WBRT—cognitive function with comparison of historical control group	42 patients with BM treated with HA-WBRT	Significantly lower decline in HVLT‑R DR (Hopkins Verbal Learning Test-Revised delayed recall) at 4 months after HA-WBRT compared to historical control. No decline in QoL. 2 patients with grade III toxicity, no grade IV/V toxicity
Brownet al. NRG-CC001 [85]	Randomized phase III Arms: 1) WBRT + memantine, 2) HA-WBRT + memantine	518 patients with brain metastases 18.5% BCBM	Median FU 7.9 months Significantly lower cognitive failure after HA-WBRT plus memantine vs. WBRT + memantine At 4 months: deterioration in executive function: 23.3% (HA-WBRT + Memantine) vs. 40.4% (WBRT + Mematine) At 6 months: Learning and memory: 11.5% (HA-WBRT + Mematine) vs. 24.7% (WBRT + Memantine)
Rodriguez et al. 2021 [81]	Randomized phase III trial Arms: 1) standard PCI, 2) HA-PCI	150 patients with brain metastases in SCLC	Median FU 40.4 months Significantly lower decline in delayed free recall in HA-PCI (23.5% vs. 5.8%) at 3 months and in total recall (47.6% vs. 14.2%) at 24 months
Yang et al. [86]	Single-blind randomized phase II trial Arms: 1) conformal WBRT, 2) HA-WBRT	65 patients with brain metastases 3.1% BCBM	Median FU 12.4 months No differences in baseline neurocognitive function. Significantly better preservation of HVLT‑R recognition–discrimination index and memory score in HA-WBRT at 6 months No differences in other cognitive tests No differences in OS or PFS
Belderbos et al. 2021 [79]	Multicenter randomized phase III trial Arms: 1) standard PCI, 2) HA-PCI	168 patients with brain metastases in SCLC	Median FU 26.6 months No significant difference in HVLT‑R total recall at 4 months (29% vs. 28%) or other cognitive tests Cumulative incidence of brain metastases at 2 years 20% vs. 16%

After SRS/SRT of BCBM, regular follow-up by a radiation oncologist with additional MRI (e.g., every 3 months) is recommended, whereas follow-up after WBRT may be guided by clinical factors. For diagnosis and treatment of radionecrosis, we refer to the DEGRO practical guideline for central nervous system radiation necrosis [87].

### Combination of radiotherapy with systemic therapy

There are two essential questions that arise in the context of combining radiotherapy and systemic therapy for BCBM:

#### 1) Are there concerns regarding increased toxicity when combining these treatments?

As most patients with BCBM are treated with systemic therapy and interruption of systemic therapy may be critical due to extracranial metastatic burden, the question of the safety of concurrent treatment combination is highly relevant. The evidence regarding toxicity of combined systemic therapy and CNS radiotherapy is sparse. Whereas the combination of endocrine therapy and SRS/WBRT is considered as safe [88]), preclinical studies have shown that the HER2-targeted antibody–drug conjugate trastuzumab emtansine (T‑DM1) may increase radiosensitivity. In a recent study involving 98 patients, Lebow et al. [89] observed that administering antibody drug–conjugates (ADC; specifically trastuzumab emtansine, trastuzumab deruxtecan [T-DXd], and sacituzumab govitecan [SG]) concurrently with stereotactic radiotherapy (either up to 7 days before or within 21 days after ADC treatment) resulted in a higher incidence of grade 4–5 higher radiation necrosis compared to non-concurrent treatments (7.1% vs. 0.7%). For previously irradiated lesions the 24-month risk of severe radionecrosis was 42.0% with and 9.4% without concurrent ADC. In univariable analysis, T‑DM1 and T‑DXd were associated with an increased risk of symptomatic radiation necrosis compared with no concurrent ADC. For the subgroup receiving SG, there was only a trend (HR 5.18, ranging from 0.64 to 42.11, *p* = 0.12) [90]. However, this subgroup was the smallest and had the broadest confidence interval. The limitations of this study include the small patient cohort, its retrospective nature, and the difficulty in distinguishing between local treatment failure and radionecrosis. While an increased risk for radiation necrosis has already been described for the combination of SRS and T‑DM1, further data are needed for T‑TXd and SG.

In other retrospective studies, SRS was well tolerated alongside CDK4/6 inhibitors [91]. Similar reports exist for immunotherapy and PARP inhibitors administered in combination with SRS [92, 93]. Data regarding the combination of trastuzumab and pertuzumab with SRS/SRT are sparse, but according to reviews and census recommendations, the combination with stereotactic radiotherapy is generally considered as safe [94, 95].

In a systematic review by Kroeze et al. investigating the toxicity of targeted therapies and stereotactic radiotherapy, high-dose SRT concurrent with targeted therapy (TT) was characterized by a favorable safety profile, regardless of whether TT was interrupted during and around SRT [96]. A consensus recommendation from the ESTRO-EORTC OligoCare Consortium published in 2023 focused on extracranial stereotactic body radiotherapy (SBRT) and systemic therapy. Consensus was reached that trastuzumab and pertuzumab can be administered on the same day as SBRT without a treatment break or dose reduction, whereas no such consensus was reached for T‑DM1. For T‑DM1, CDK4/6 inhibitors, HER2 inhibitors, and PARP inhibitors, there was consensus that SBRT can be performed without dose reduction; however, there was no consensus regarding interruption of systemic therapy during SBRT. These recommendations should not be uncritically applied to SRS/SRT for BCBM, since pathomechanisms of toxicity may differ [94]. Due to the lack of reliable data, the decision on whether systemic therapy needs to be interrupted during radiotherapy should be made individually on a case-by-case basis within an interdisciplinary board.

#### 2) Can local therapy be postponed when systemic therapy with relevant intracranial response rates is given?

Local interventions such as resection and radiation are the standard of care for BCBM, but systemic therapies are increasingly effective in the treatment of brain metastases. In patients with HER2+ BCBM, relevant intracranial response rates have been reported for antibody–drug conjugates like T‑DM1 and T‑DXd as well as kinase inhibitor-containing regimens such as tucatinib/capecitabine/trastuzumab, neratinib/capecitabine, and lapatinib/capecitabine [97, 98]. The intracranial response rates in some of the randomized studies are reported to be > 60% in treatment-naïve HER2+ BCBM. The randomized controlled HER2CLIMB trial demonstrated an improvement in overall survival in the predefined subgroup of 291 patients with BCBM with the addition of tucatinib to trastuzumab/capecitabine. In this trial, 174 patients were considered to have active brain metastases; however, only 66 of these patients had previously untreated BCBM (Table 6).

**Table 6:** Studies investigating the role of systemic therapies in patients with HER2+ metastatic breast cancer

Study	Design	Study collective/proportion of BCBM	Results
*Systemic therapies in HER2+ metastatic BC*			
EMILIA (Krop, Lin et al. 2015 [97])	Retrospective, exploratory analysis of the EMILIA trial Arms: T‑DM1 vs. XL after trastuzumab therapy	95 patients with treated, asymptomatic HER2+ BCBM. 45 patients received T‑DM1, 50 patients received XL	Improved median OS with T‑DM1 in patients with BCBM at baseline (26.8 vs. 12.9 months), similar PFS in both arms (5.9 vs. 5.7 months) There was less grade ≥ III toxicity with T‑DM1 compared to XL
HER2Climb (Lin, Murthy et al. 2023 [99])	Preplanned subgroup analysis of the HER2CLIMB trial Arms: tucatinib + trastuzumab/capecitabine vs. placebo + trastuzumab/capecitabine	291 patients with HER2+ BCBM, 66 patients with untreated BCBM	Prolonged median OS with Tucatinib (21.6 vs. 12.5 months) and intracranial PFS (13.9 vs. 5.6 months) Also improved intracranial overall response rate (ORR) with tucatinib (47.3% vs. 20.0%)
Destiny 03 (Hurvitz, Hegg et al. 2023 [100])	Subgroup analysis of a randomized phase III study Arms: T‑DM1 vs. T‑DXd	82 patients with treated, asymptomatic BCBM at baseline (43 patients treated with T‑DXd and 39 patients treated with T‑DM1)	Consistent OS benefit in favor of T‑DXd across subgroup analyses, including those with BM
TUXEDO‑1 (Bartsch, Berghoff et al. 2022 [101])	Single-center prospective single-arm phase II study on T‑DXd	15 patients with HER2+ BCBM, in 40% BCBM were previously untreated	ORR of 73.3%, median PFS of 14.0 months
KAMILLA (Montemurro, Delaloge et al. 2020 [102])	Multicenter prospective single-arm study on T‑DM1	398 patients (19.9%) with BCBM, 56.8% of whom had received RT previously	Median PFS/OS was 5.5/18.9 months in patients with baseline BM. Best intracranial ORR was 21.4%
LANDSCAPE (Bachelot, Romieu et al. 2013 [103])	Multicenter prospective single-arm study on lapatinib + capecitabine	45 patients with HER2+ previously untreated BCBM	Median OS 17.0 months. Intracranial ORR 66%. 49% with grade III/IV treatment-related toxicity
NALA (Saura, Oliveira et al. 2020 [104])	Subgroup analysis of a multicenter randomized controlled phase III study Arms: neratinib + capecitabine (N + C) vs. lapatinib + capecitabine (L + C)	101 patients with HER2+ BCBM (stable and asymptomatic at baseline)	No improvement in PFS and OS. Significant improvement of intracranial ORR with N + C (26.3% vs. 15.4%)
TBCRC-022 (Freedman, Gelman et al. 2016 [105])	Multicenter single-arm phase II study on neratinib	40 patients with HER2+ BCBM after WBRT/SRS/surgery	Median OS was 8.7 months, median PFS was 1.9 months. Intracranial ORR was 8%
PATRICIA (Lin, Pegram et al. 2021 [106])	Multicenter single-arm phase II study on neratinib/capecitabine	49 patients with HER2+ BCBM	Outcome in lapatinib-naïve patients: median OS was 13.3 months, median PFS was 5.5 months. Intracranial ORR was 49%
NEfERT‑T (Awada, Colomer et al. 2016 [107])	Multicenter single-arm phase II study on pertuzumab + high-dose trastuzumab	39 patients with HER2+ BCBM previously treated with WBRT/SRS	Intracranial ORR was 11%, 6 months clinical benefit rate (CR/PR/SD) 51%, grade ≥ III toxicity in 44% of patients, no grade V toxicity

*T-DM1* trastuzumab-emtansine, *XL* capecitabine, *BCBM* breast cancer brain metastasis, *OS* overall survival, *PFS* prrogression-free survival, *ORR* overall response rate, *T-DXd* trastuzumab deruxtecan, *BM* brain metastasis, *RT* radiotherapy, *N+C* neratinib + capecitabine, *L+C* lapatinib + capecitabine, *WBRT* whole brain radiotherapy, *SRS* stereotactic radiosurgery, *CR* complete response, *PR* partial response, *SD* stable disease

Hence, when interpreting these data, it is crucial to consider the inclusion criteria of the underlying trials. While most trials enrolled patients with small asymptomatic brain metastases not requiring immediate local therapy, only a minority allowed for active brain metastases that were either untreated or progressed after prior local therapy. Furthermore, high intracranial response rates must be interpreted in the context of progression-free and overall survival as well as toxicity. Trials comparing systemic therapy only vs. systemic therapy with SRS/SRT are missing so far in HER2+ BCBM. Data regarding the treatment of HER2− (luminal or TNBC) BCBM are even more sparse. For the CDK4/6 inhibitor abemaciclib, an intracranial response (defined as complete or partial response) was observed in 5.2% of patients, median intracranial PFS was 4.9 months [108]. According to a small phase II trial, brain metastases in primary tumors with a PD-L1 of ≥ 1% showed in 29.7% a response to pembrolizumab [109].

It should be noted that local control rates of SRS with up to > 90% after 2 years, are higher than the response rates in any of the existing trials on systemic therapy for BCBM. Nevertheless, intracranial control is an issue with SRS/SRT, as 30–40% of patients experience distant intracranial progression within 1 year. Furthermore, it needs to be considered, as mentioned earlier, that the receptors in brain metastases differ from the primary tumor in more than 40% of patients. Given the high risk of complications in case of progressive brain metastases and the low rate of toxicity of SRS/SRT, local therapy is generally recommended in limited BCBM regardless of molecular subtype and systemic therapy. If SRS/SRT is technically infeasible, in patients with multiple (≥ 5) asymptomatic HER2+ BCBM (e.g., due to prior irradiation), systemic therapy combined with early reassessment of response (after 8–12 weeks) and reevaluation of radiotherapy can be considered after interdisciplinary discussion to defer or avoid WBRT. Similarly, in case of asymptomatic disseminated brain metastases, systemic therapy combined with early reassessment can be considered after interdisciplinary discussion. However, in case of systemic therapy only, patients need to be informed about the risk of progressive intracranial disease with subsequent complications and should be actively involved in the decision.

## Recommendations

- The biological subtype and breast cancer-specific GPAs should be considered for evaluation of prognosis in patients with BCBM.
- Limited brain metastases (*n* = ≤ 4):
  - Local therapy including SRS/SRT is generally recommended irrespective of molecular subtype and systemic therapy.
  - In case of limited intact BCBM (*n* = ≤ 4), SRS/SRT should be used.
  - After resection with a limited number of remaining BCBM (*n* = ≤ 4), SRS/SRT to the resection cavity should be used as postoperative treatment with additional SRS/SRT of the intact BCBM.
- Multiple brain metastases:
  - SRS should be considered in case of *n* = 5–10 intact BCBM (cumulative volume < 15 ml); alternatively, WBRT can be applied.
  - After resection of BCBM and limited further BCBM (*n* = 5–10 and < 15 ml), SRS/SRT to the resection cavity and remaining intact BCBM is a possible option. Alternatively, WBRT can be applied.
  - In disseminated brain metastases (*n* = > 10), WBRT is generally recommended.
  - After interdisciplinary discussion, in cases of asymptomatic disseminated brain metastases (*n* = > 10) or in multiple BCBM if SRS/SRT is not feasible, WBRT can be postponed with early reassessment and reevaluation of local treatment options (8–12 weeks) if HER2-targeted systemic therapy with significant response rates in the CNS (tucatinib/trastuzumab/capecitabine, trastuzumab deruxtecan) is being used.
- Leptomeningeal carcinomatosis:
  - In symptomatic leptomeningeal carcinomatosis, local radiotherapy (WBRT/involved-field SRS/SRT or local spinal irradiation) should be administered to symptomatic lesions in addition to systemic therapy.
  - In case of patients with disseminated leptomeningeal carcinomatosis in good clinical condition and with limited, stable extra-CNS disease, CSI may be considered.
- Technique:
  - The decision on the optimal fractionation regimen is dependent on the size and location of the metastases/resection cavity.
  - In case of WBRT, hippocampal avoidance should be considered, especially for patients with a good prognosis according to GPA score.
  - Administration of memantine or donepezil may be considered with WBRT (off-label use in Germany).
- Concurrent systemic therapy:
  - There is a general lack of data regarding the combination of systemic therapy and SRS/SRT for brain metastases.
  - Each case should be discussed individually in an interdisciplinary setting based on the type of systemic therapy, size and location of the metastases, as well as planned dose and fractionation.
  - Particular caution should be taken when administering SRS/SRT concurrently (≤ 7 days before or ≤ 21 days after) with antibody drug–conjugates.

## Conclusion

Due to the increasing effectiveness of systemic therapy in improving long-term survival in metastatic breast cancer, achieving local control in the CNS has become a crucial treatment goal. SRS and fractionated SRT have demonstrated excellent control rates and low toxicity rates for cases with a limited number of brain metastases, regardless of molecular subtype and systemic therapy, and should therefore be recommended. For patients with leptomeningeal carcinomatosis, local radiation can significantly improve symptoms. In cases with favorable prognostic factors, CSI may also be performed to improve oncological outcomes.
